# Demonstration of quantum permutation algorithm with a single photon ququart

**DOI:** 10.1038/srep10995

**Published:** 2015-06-05

**Authors:** Feiran Wang, Yunlong Wang, Ruifeng Liu, Dongxu Chen, Pei Zhang, Hong Gao, Fuli Li

**Affiliations:** 1 MOE Key Laboratory for Nonequilibrium Synthesis and Modulation of Condensed Matter, and Department of Applied Physics, Xi'an Jiaotong University, Xi'an 710049, China

## Abstract

We report an experiment to demonstrate a quantum permutation determining algorithm with linear optical system. By employing photon's polarization and spatial mode, we realize the quantum ququart states and all the essential permutation transformations. The quantum permutation determining algorithm displays the speedup of quantum algorithm by determining the parity of the permutation in only one step of evaluation compared with two for classical algorithm. This experiment is accomplished in single photon level and the method exhibits universality in high-dimensional quantum computation.

As quantum counterpart of classical computer, quantum computer reveals incredible efficiency to execute arithmetic tasks and threatens the security of classical communication. Quantum algorithm is the sole of quantum computation, which shows the amazing power of quantum parallelism and quantum interference. It attracts particular concern to develop new quantum algorithms in recent years. The concept of simulating physics progresses with quantum computers was originated in Richard Feynman's observation that computers built from quantum mechanical components would be ideally suited to simulating quantum mechanics^1^. Since then, the first efficient quantum algorithm was proposed by Deutsch in 1985^2^ and generalized by Deutsch and Jozsa in 1987^3^. Lately, an increasing number of practical programs were presented, such as factoring large integer^4^, Grover's searching algorithm for database^5^ and Simon's exponential acceleration algorithm for the black box problem^6^. What's more, Harrow *et al.* came up with a quantum scheme to decrease the computational complexity of solving linear system of equations from *O*(*n*) to log(*n*) , and this was the first quantum algorithm to work out the most fundamental problems in engineering science^7^. Some quantum algorithms have been demonstrated in different physical systems, such as ion traps^8^,^9^,^10^,^11^, superconducting devices^12^,^13^,^14^, optical lattices^15^,^16^, quantum dots^17^,^18^, and linear optics^19^,^20^,^21^,^22^,^23^,^24^,^25^. Due to its good scalability, easy-handling and high stability, linear optical system is a good candidate for implementing quantum algorithms.

There is a permutation problem which is to determine the parity of the permutation realized by a black box. For example, considering a black box to realize permutation operation *f*(*x*) on a input set *x* ∈ {1, 2, 3}, the output states have six different possible permutations (1, 2, 3), (2, 3, 1), (3, 1, 2), (3, 2, 1), (2, 1, 3) and (1, 3, 2), where the first three are positive cyclic or even permutations and the last three are negative cyclic or odd permutations. To determine the parity of transformation requires the evaluation of *f*(*x*) for at least two different input values of *x* with classical algorithm. However, a new algorithm based on quantum Fourier transformation has been proposed recently to solve this problem with only one step of evaluation^26^.

In this report, we consider four orthonormal states, i.e., 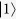 = (1, 0, 0, 0)^*T*^, 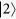 = (0, 1, 0, 0)^*T*^, 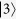 = (0, 0, 1, 0)^*T*^, and 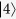 = (0, 0, 0, 1)^*T*^, and the bijection is *f* : {1, 2, 3, 4} → {1, 2, 3, 4}. For the input state (1, 2, 3, 4), there are eight different possible output states. These eight output states and the corresponding transformations are divided into two categories as mentioned above. The positive cyclic permutations and the corresponding unitary transformations are:
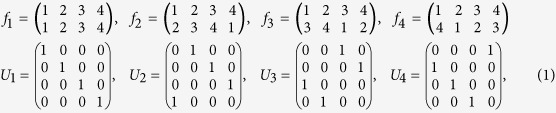


and the negative cyclic permutations and the corresponding unitary transformations are:
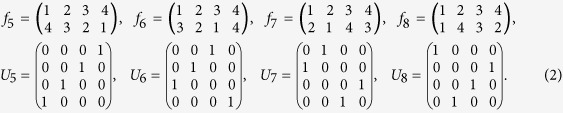


The black box can be regarded as a special device which can realize the corresponding operation for a given permutation task.

To discriminate the parity, a direct running of the operator on the eigen states of ququart will not work. For example, if we input a state 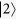 and get the output state 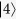, this progress corresponds to two possible permutation transformations *f*_3_ and *f*_8_. Therefore at least twice runnings are needed to evaluate the box as in the classical case.

The quantum circuit to realize the permutation algorithm is shown in Fig. 1. Considering the quantum algorithm of this task, we start from a superposition state
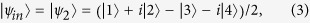
here 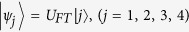, and *U*_*FT*_ is the quantum Fourier transformation:
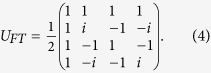


Then the input state gets through the black box, and the output state can be written as
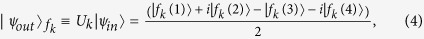
where *k* = 1, 2,…, 8 labels eight permutation transformations. The output state will reveal the parity of *f*_*k*_—even for 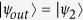 and odd for 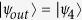. By employing the inverse Fourier transform 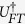 on 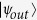 and checking the final state, we will know the parity of permutation is even (odd) when we acquire the state 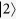

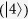
. Thus, the quantum algorithm allows us to determine the parity of a cyclic permutation with only single evaluation of the permutation function instead of two.

Reviewing this algorithm, the Fourier transform *U*_*FT*_ and inverse Fourier transform 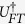 are not necessary if we can directly prepare the superposition state 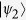 and discriminate the output state 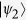 and 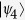. Luckily, by employing the photon's polarization and spatial mode, we can easily realize the ququart 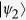 and distinguish 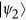 and 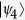 in our experiment. We should address that 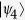 is also a proper input state for the permutation discrimination, and in our experiment, we have demonstrated the algorithm for both 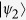 and 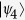.

## Results

In our experiment, we utilize photon's polarization and spatial mode to code the ququart^23^, and we carry out the whole eight different permutations in the black box which is composed by Dove prism (DP) and half wave plates (HWP). The four eigen states of ququart can be defined as
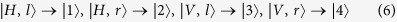
where *H*(*V*) represents horizontal (vertical) polarization and *r*(*l*) represents right (left) spatial mode. Thus the input state 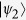 can be written in the form
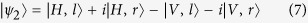


Then the black box will carry out the permutation transformation on 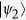. For example, suppose the operation is *f*_2_, the output state after the black box can be expressed as 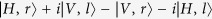, which is equal to 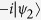.

**Figure 2:**
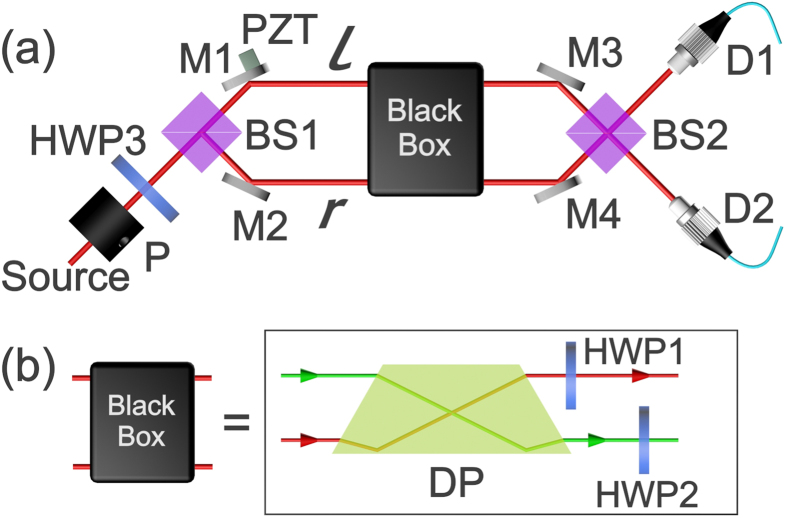
Experimental setup of permutation algorithm. (**a**) The single photon source is achieved by deeply attenuating coherent light into single photon level. P denotes the polarizer for preparing horizontal polarization state. Half wave plate (HWP) is used for initial polarization state preparation. Two beam splitters (BS) and four mirrors (M) are used to set up a Mach-Zehnder interferometer. The piezo transmitter (PZT) is used to modulate the phase *φ* between *l* and *r* paths. The black box is used to realize eight permutation transformations. Two detectors (D1 and D2) are single-photon counting modules (SPCM-AQRH-14-FC) used to record the count of photons. (**b**) The black box consists of a DP at 0^°^ and two HWPs at 45^°^. Different permutation transformation can be achieve by different combination of DP and HWPs.

The sketch of the experimental setup is shown in Fig. 2(a). The source is achieved by deeply attenuating coherent light into single photon level (coherent parameter *α* ≈ 0.1). Polarizer (P) and HWP3 are placed to prepare the initial input polarization state and beam splitter (BS1) is to prepare spatial state. The black box consists of a DP and two HWPs as shown in Fig. 2(b), the dove prism is located at an angle 0^°^ relative to the horizontal plane. The spatial modes (*r* and *l*) and polarizations (*H* and *V*) will be swapped by DP at 0^°^ and HWP at 45^°^, respectively. By adding or removing the DP or HWPs, all the permutation transformations can be achieved succinctly.

In this scheme, we prepare the initial polarization state of photon in a superposition state 
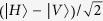
, then we transform the spatial mode into 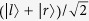 by BS1. The relative phase *φ* between two spatial modes can be adjusted by a PZT mounted on the first reflecting mirror (M1). Thus the input state can be written as
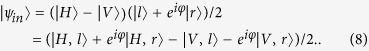
Especially, when *φ* = *π*/2, the initial state 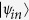 turns to be 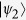 which is expressed in Eq (7); when *φ* = 3*π/*2, the initial state 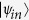 turns to be 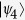. Then the state is injected into the black box to carry out the permutation transformations. The states of 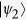 and 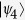 in our definition can be discriminated directly by checking the phase in spatial mode . So we let the different spatial states interfere at the second beam splitter (BS2), and the parity of permutation can be determined by checking which single photon detector (D1 or D2) clicks.

### Implementation of positive cyclic permutation

For the identity operation *f*_1_, we remove the DP and HWPs, the input states pass through the setup remain unchanged. For the *f*_2_ operation, DP and HWP1 are required in the setup. When the photons in different paths pass through the DP, they will exchange their paths (*l* → *r* and *r* → *l*), and the state 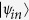 changes into 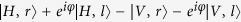. After that, HWP1 at an angle 45^°^ is located at the *l* path, and it will alter horizontal polarization to vertical polarization and vice versa. So far we get the final state 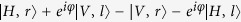 which is equal to 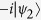 when *φ = π*/2 and achieve the mapping operation *f*_2_. The *f*_3_ operation can be carried out similarly by removing the DP and inserting HWP1 and HWP2. Analogous to the action above, the last positive cyclic permutation *f*_4_ can be accomplished by inserting DP and HWP2. When photons pass through the device for the positive cyclic permutation, a simple calculation shows the output state 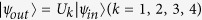 as follows:
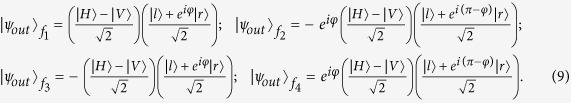


### Implementation of negative cyclic permutation

*f*_5_ can be realized by placing both DP and HWPs into the optical route. If we remove the DP and only place HWP1, when the photons pass this setup, it will go through an *f*_6_ transformation. For the purpose of carrying out *f*_7_ operation, it is easy to obtain that only a DP is needed. The last operation can be achieved by only employing HWP2 to change the polarization mode on the right route. Similarly, we arrive the final output states:
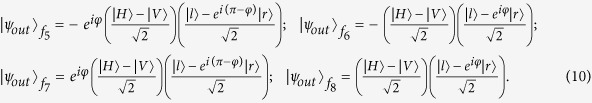


From the above discussion, we carry out all the eight essential transformations for the parity determining algorithm. All the transformations and corresponding implementation approaches are summarized in Table 1. In particular, when 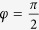, the output states in Eqs. (9) and (10) are equal to 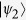 and 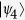, respectively. The Eqs. (9) and (10) clearly show that, for different parity of permutations, the polarization of output states are same while the relative phase between two spatial modes are different. This means that we can accomplish the progress of identify only on the spatial qubits and save the inverse Fourier transformation. After the spatial modes interfered on BS2, we can determine the parity is odd (even) when detector D1 (D2) clicks. With similar analysis, when the input state is 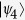 (relative phase 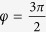), we can determine the parity is odd (even) when detector D2 (D1) clicks.

**Figure 3:**
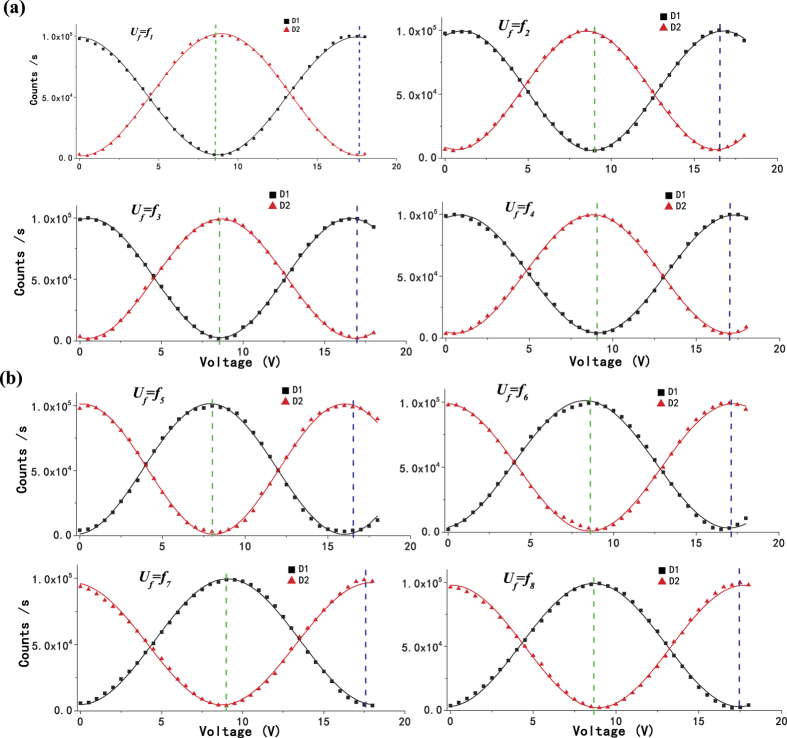
Experimental data of the parity determining algorithm. (**a**) positive cyclic permutation operation, (**b**) negative cyclic permutation operation. Black square dots show the photon counts of D1, and red triangle points show the photon counts of D2. Fitting lines are also displayed. Green (blue) dashed vertical lines are used to mark the proper phases of the initial states 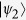 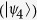, which can be used to perfectly discriminate the parity of transformations.

We record the photon counts of the two detectors D1 and D2 with 0.5 V a step of the PZT voltage synchronously. Our experimental results are shown in Fig. 3. Figure 3(a) shows the results of positive cyclic permutation transformations from *f*_1_ to *f*_4_, and Fig. 3(b) gives the results of the remaining four odd operations. The black square spots represent the counts of D1, and the red triangular spots corresponding to the counts of D2. As we discussed above, when the relative phase *φ* is equal to (2*N* + 1/2)*π*, where N is an integer, only detector D1 clicks for the odd parity and D2 clicks for the even parity. These special points are pointed out by the green dashed line, and from these points we can get the parity information of the permutation evidently. Analogously, when the relative phase *φ* is equal to (2*N* + 3/2)*π*, we still can determine the parity by the blue dashed line labelled in Fig. 3.

## Discussion

We define 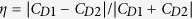 as the contrast ratio to evaluate the accuracy of our experiment, where *C*_*D*1_ and *C*_*D*2_ denote the photon counts of D1 and D2, respectively. Theoretically, the contrast ratio *η* is equal to 1. In our experiment, the contrast ratio is 96 ± 2% for all eight cases. The error and the small shift of the dashed line for different permutations mainly comes from the imperfection of optical devices and removing or adding DP and HWPs.

In conclusion, we briefly introduce the quantum permutation algorithm and put forward a scheme to implement this algorithm by employing linear optical elements . By using composite qubit to realize the ququart, our experiment is greatly simplified both in state preparation and state discrimination, where Fourier transformations *U*_*FT*_ and 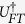 are saved. To our knowledge, this is the first time of realization quantum permutation algorithm in linear optical system. The experimental method we used is simple and pave a way for realization other high-dimensional quantum information tasks. Although this algorithm only provide a two to one speed-up towards classical case, it shows the power of quantum parallelism and quantum computation validly. As we know, generating a new quantum algorithm is quite difficult. We wish this algorithm can expand our thinking for more efficient algorithms. We noticed that this scheme has also been realized in spin-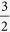 NMR quadrupolar system with four energy levels^27^,^28^.

## Method

In this experiment, we used a typical Mach-Zehnder interferometer to realize the determination of permutation. The interferometer was located on a special small experimental platform and had a soft buffer to resist the vibration noise. And the experimental elements were placed as compact as possible to make the interferometer more stable. The Mach-Zehnder interferometer was stable enough for us to accomplish the data measurement. Furthermore, we also calibrated the voltage of PZT and the initial phase between two paths each time before inserting or removing the optical components.

For the specific operation such as *f*_2_, only one HWP at 45^°^ is needed in the left route, therefore this HWP gives an additional phase because of the intrinsic thickness. With the purpose of eliminating this extra effect, we put another HWP at 0^°^ in the right path to compensate phase without changing the polarization.

## Additional Information

**How to cite this article**: Wang, F. *et al.* Demonstration of quantum permutation algorithm with a single photon ququart. *Sci. Rep.* **5**, 10995; doi: 10.1038/srep10995 (2015).

## Figures and Tables

**Figure 1:**
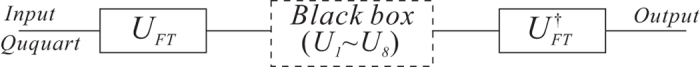
Quantum circuit to realize the permutation algorithm. *U*_*FT*_ denotes the Fourier transformation. The black box contains all the eight permutation operators in the algorithm and 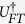 is the inverse Fourier transformation.

**Table 1:**
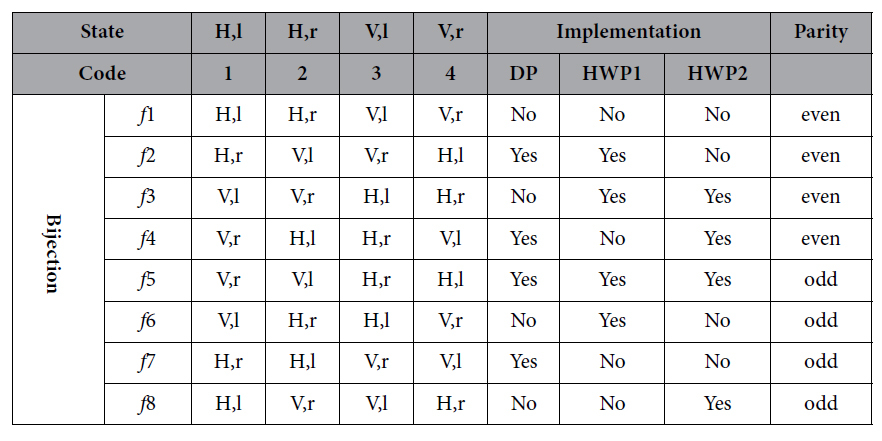
Experimental implementation of eight different permutations.

## References

[b1] Feynman R. P. Simulating physics with computers. Int. J. Theor. Physics. 21, 467–488 (1982).

[b2] Deutsch D. Quantum theory, the Church-Turing principle and the universal quantum computer. Proc. R. Soc. Lond. A. 400, 97–117 (1985).

[b3] Deutsch D. & Jozsa R. Rapid solution of problems by quantum computation. Proc. R. Soc. A 439, 553 (1992).

[b4] Shor P. W. Algorithms for quantum computation: discrete logarithms and factoring. IEEE Comp. Soc. 11, 124–134 (1994).

[b5] Grover L. K. Quantum mechanics helps in searching for a needle in a haystack. Phys. Rev. Lett. 79, 325 (1997).

[b6] Simon D. R. On the power of quantum computation. SIAM J. on Computing 26, 116–123 (1994).

[b7] Harrow A. W., Hassidim A. & Lloyd S. Quantum algorithm for linear systems of equations. Phys. Rev. Lett. 103, 150502 (2009). 19905613 10.1103/PhysRevLett.103.150502

[b8] Cirac J. I. & Zoller P. Quantum computations with cold trapped ions. Phys. Rev. Lett. 74, 4091 (1995). 10058410 10.1103/PhysRevLett.74.4091

[b9] Blatt R. & Wineland D. Entangled states of trapped atomic ions. Nature 453, 1008–1015 (2008). 18563151 10.1038/nature07125

[b10] Lanyon B. P. *et al.* Universal digital quantum simulation with trapped ions. Science 334, 57 (2011). 21885735 10.1126/science.1208001

[b11] Lanyon B. P. *et al.* Measurement-based quantum computation with trapped ions Phys. Rev. Lett. 111, 210501 (2013). 24313469 10.1103/PhysRevLett.111.210501

[b12] Nakamura Y., Pashkin Y. A. & Tsai J. S. Coherent control of macroscopic quantum states in a single-Cooper-pair box. Nature 398, 786–788 (1999).

[b13] DiCarlo L. *et al.* Demonstration of two-qubit algorithms with a superconducting quantum processor. Nature 460, 240–244 (2009). 19561592 10.1038/nature08121

[b14] Bialczak R. C. *et al.* Quantum process tomography of a universal entangling gate implemented with Josephson phase qubits. Nat. Phys. 6, 409 (2010).

[b15] Jaksch D. Optical lattices, ultracold atoms and quantum information processing. Contemp. Phys. 45, 367 (2004).

[b16] Bloch I. Quantum coherence and entanglement with ultracold atoms in optical lattices. Nature 453, 1016–1022 (2008). 18563152 10.1038/nature07126

[b17] Berezovsky J., Mikkelsen M. H., Stoltz N. G., Coldren L. A. & Awschalom D. D. Picosecond coherent optical manipulation of a single electron spin in a quantum dot. Science 320, 349–352 (2008). 18420929 10.1126/science.1154798

[b18] Fushman I. *et al.* Controlled phase shifts with a single quantum dot. Science 320, 769–772 (2008). 18467584 10.1126/science.1154643

[b19] Knill E., Laflamme R. & Milburn G. J. A scheme for efficient quantum computation with linear optics. Nature 409, 46–52 (2001). 11343107 10.1038/35051009

[b20] O'Brien J. L., Pryde G. J., White A. G., Ralph T. C. & Branning D. Demonstration of an all-optical quantum controlled-NOT gate. Nature 426, 264–267 (2003). 14628045 10.1038/nature02054

[b21] Mohseni M., Lundeen J. S., Resch K. J. & Steinberg A. M. Experimental application of decoherence-free subspaces in an optical quantum-computing algorithm. Phys. Rev. Lett. 91, 187903 (2003). 14611316 10.1103/PhysRevLett.91.187903

[b22] Lu C.-Y., Browne D. E., Yang T. & Pan J.-W. Demonstration of a compiled version of Shor's quantum factoring algorithm using photonic qubits. Phys. Rev. Lett. 99, 250504 (2007). 18233508 10.1103/PhysRevLett.99.250504

[b23] Zhang P., Liu R. F., Huang Y. F., Gao H. & Li F. L. Demonstration of Deutsch's algorithm on a s table linear optical quantum computer. Phys. Rev. A 82, 064302 (2010).

[b24] Cai X.-D. *et al.* Experimental quantum computing to solve systems of linear equations. Phys. Rev. Lett. 110, 230501 (2013). 25167475 10.1103/PhysRevLett.110.230501

[b25] Tame M. S., Bell B. A., Di Franco C., Wadsworth W. J. & Rarity J. G. Experimental realization of a one-way quantum computer algorithm solving simon's problem. Phys. Rev. Lett. 113, 200501 (2014). 25432032 10.1103/PhysRevLett.113.200501

[b26] Gedik Z. Computational speed-up with a single qutrit. e-print: arXiv:1403.5861 (2014).

[b27] Dogra S., Arvind & Dorai, K. Determining the parity of a permutation using an experimental NMR qutrit. Phys. Lett. A 378, 3452 (2014).

[b28] Silva I. A. *et al.* Computational speed-up in a single qudit NMR quantum information processor. e-print: arXiv:1406.3579 (2014).

